# Expression Profiling and Bioinformatics Analysis of CircRNA in Mice Brain Infected with Rabies Virus

**DOI:** 10.3390/ijms22126537

**Published:** 2021-06-18

**Authors:** Wen Zhao, Jingyin Su, Ningning Wang, Naiyu Zhao, Shuo Su

**Affiliations:** College of Veterinary Medicine, Nanjing Agricultural University, Nanjing 210095, China; 2017207031@njau.edu.cn (W.Z.); xiao_anny@yeah.net (J.S.); 17416104@njau.edu.cn (N.W.); 17117420@njau.edu.cn (N.Z.)

**Keywords:** rabies virus, mice brain, circRNAs, enrichment analysis, ceRNA network

## Abstract

Rabies virus (RABV) induces acute, fatal encephalitis in mammals including humans. The circRNAs are important in virus infection process, but whether circRNAs regulated RABV infection remains largely unknown. Here, mice brain with or without the RABV CVS-11 strain were subjected to RNA sequencing and a total of 30,985 circRNAs were obtained. Among these, 9021 candidates were shared in both groups, and 14,610 and 7354 circRNAs were expressed specifically to the control and experimental groups, indicating that certain circRNAs were specifically inhibited or induced on RABV infection. The circRNAs mainly derived from coding exons. In total, 636 circRNAs were differentially expressed in RABV infection, of which 426 significantly upregulated and 210 significantly downregulated (*p* < 0.05 and fold change ≥2). The expression of randomly selected 6 upregulated and 6 downregulated circRNAs was tested by RT-qPCR, and the expression trend of the 11 out of 12 circRNAs was consistent in RT- qPCR and RNA-seq analysis. Rnase R-resistant assay and Sanger sequencing were conducted to verify the circularity of circRNAs. GO analysis demonstrated that source genes of all differentially regulated circRNAs were mainly related to cell plasticity and synapse function. Both KEGG and GSEA analysis revealed that these source genes were engaged in the cGMP–PKG and MAPK signaling pathway, and HTLV-I infection. Also, pathways related to glucose metabolism and synaptic functions were enriched in KEGG analysis. The circRNA–miRNA–mRNA network was built with 25 of 636 differentially expressed circRNAs, 264 mRNAs involved in RABV infection, and 29 miRNAs. Several miRNAs and many mRNAs in the network were reported to be related to viral infection and the immune response, suggesting that circRNAs could regulate RABV infection via interacting with miRNAs and mRNAs. Taken together, this study first characterized the transcriptomic pattern of circRNAs, and signaling pathways and function that circRNAs are involved in, which may indicate directions for further research to understand mechanisms of RABV pathogenesis.

## 1. Introduction

Rabies is a zoonotic viral disease that has threatened human life for centuries. Approximately 59,000 human deaths are reported annually worldwide, especially in Africa and Asia (https://www.who.int/, accessed 6 May 2021). Although rabies is vaccine-preventable, no effective treatment exists once clinical symptoms develops. The viral proteins of RABV inhibit the host’s antiviral innate immune response through multiple mechanisms. The M protein of RABV inhibits the expression of antiviral cytokines (such as HIAP1, IRF1, IFN-β, and TNF), and induces CXCL2 and IL8 expression by interacting with RelAp43 [1,2]. The N protein of virulent strain poorly induces the activation of retinoic acid-inducible gene 1 protein (RIG-I) and the expression of IFN-β, CXCL10, and CCL5 [3]. The P protein impairs the phosphorylation of interferon regulator factor 3 (IRF3) and interferon regulator factor 7 (IRF7) and the transcription of IFN-β mRNA to promote viral invasion [4,5]. The nuclear transport of STAT1 was impeded by P protein, thus inhibiting the host immune response. Moreover, the P protein weakens the innate immune response by modifying nuclear bodies structure and destroying the antiviral effect of PML [6,7]. Also, RABV induces autophagy by interacting with BECN1 via the CASP2-AMPK-MAPK/AKT-MTOR pathway [8] and induces apoptosis [9] to promote self-replication. Host proteins are necessary in pathogenicity of RABV. The upregulation of host FASL, HLA-G and B7-H1 obstructs host antiviral responses and facilitates virus infection by regulating T cell proliferation or apoptosis [10,11,12]. Immune-related molecular, such as Viperin [13], Toll-like receptor 3 (TLR3) [14], Toll-like receptor 7 (TLR7) [15], and IFN-stimulated genes such as IFITM3 [16], IFIT2 [17], and ISG15 [18], play important roles in host anti-RABV responses. Recently, GTPase 1, an interferon-inducible gene, was proven to restrict RABV replication by blocking the dimerization of P protein [19]. Although many studies have been conducted, the pathogenesis of rabies virus has not yet been fully understood. Insufficient understanding of its pathogenesis mechanism restricts the improvement of treatment methods and technology for rabies.

Circular RNAs (circRNAs) are non-coding RNAs and show resistance to exonuclease digestion due to a lack of 3′ and 5′ ends, and thus are more stable than linear transcripts. The circRNAs are widely present in the eukaryotic transcriptome, but usually at low levels with regulatory functions [20,21]. CircRNAs in the nuclear may regulate transcription and alternative splicing of their parent genes [22,23,24]. In the cytoplasm, many circRNAs bind miRNAs to regulate function of the miRNA target genes [20,21,25,26]. Some circRNAs interact with RNA-binding proteins (RBPs) to inhibit host genes translation or to regulate cell process [27,28]. Although circRNAs are commonly regarded as non-coding RNAs, some of them have shown that they regulate tumor process through translating into peptides [29,30]. In fact, circRNAs were reported to be involved in various human diseases via the regulation of autophagy [31,32], apoptosis [33], or cell cycle progression [28], making them potential candidates of clinical diagnosis markers and therapeutic agents [34,35]. Additionally, circRNAs participate in the host antiviral immune response [36]. The upregulated expression of circ-Vav3 in tumor caused by Avian leukosis virus subgroup J (ALV-J) promotes viral infection via sponging gga-miR-375 [37]. Influenza A virus (IAV) H1N1 induces circ-GATAD2A expression and ectopic expression of circ-GATAD2A promotes H1N1 replication through impairing autophagy [38]. Collectively, circRNAs are involved in virus infection, although their function is still largely unknown.

Here, we used RABV-infected or non-infected mice brains to investigate circRNAs profiling changes using RNA sequencing. We identified and characterized the expression profiling of circRNAs, conducted functional enrichment analysis of source genes of differentially expressed circRNAs, and constructed the circRNA–miRNA–mRNA network. This study may give new insights into the RABV–host interactions and pathogenic mechanisms of RABV infection.

## 2. Results

### 2.1. Validation of RABV Infection in Mice Brains

**Figure 1:**
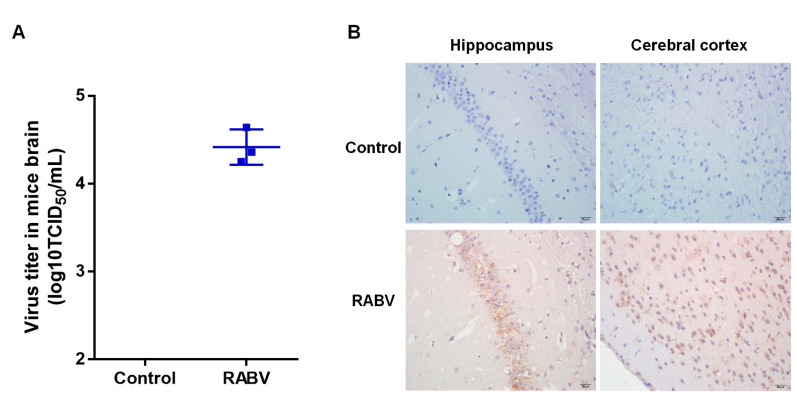
Establishment of RABV mouse infection model. (**A**) Quantification of infectious virus titer in mice brain by indirect immunofluorescence assay (IFA); (**B**) Immunohistochemistry (IHC) analysis of the cerebral cortex and hippocampus to visualize RABV infection using anti-N antibody. Scale bar = 20 µm.

In order to analyze the changes of circRNAs expression induced by RABV infection, we infected mice with the CVS-11 strain of RABV and we infected the control group of mice with DMEM. Both groups were monitored daily. In comparison with the control group, the experimental group lost weight and behaved with ruffled fur, were depressed and even displayed paralysis. Mice in both groups were euthanized, and then brain samples were collected. The viral titer was examined by the Reed–Muench method. We found that the virus titer was 10^4^ TCID_50_/mL in the experimental group, while no virus was detected in the control group (Figure 1A). To visualize RABV infection in the brain, immunohistochemical (IHC) assay was performed. RABV-infected mice brains showed extensive infection with detectable viral N protein in the cerebral cortex and hippocampus, whereas no viral proteins were observed in the control group samples (Figure 1B). Taken together, RABV successfully infects and replicates in mice brains.

### 2.2. Characteristics of circRNAs Expressed in Mice Brain by RNA Sequencing

**Figure 2:**
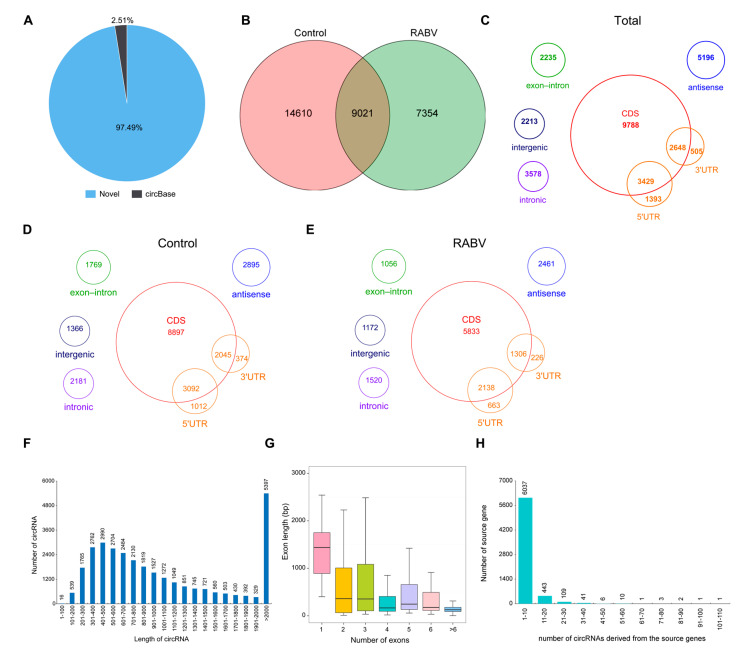
Profiling of circular RNAs in RABV- and DMEM-infected mice brains. (**A**) Pie chart showing the number of known circRNAs in Circbase and the new identified circRNAs; (**B**) The total number of circRNAs detected in the experimental and control groups; (**C**) Genomic features of circRNAs in both groups; (**D**) Genomic features of circRNAs in the control group; (**E**) Genomic features of circRNAs in the experimental group; (**F**) Illustration of the identified circRNAs length; (**G**) Box plot illustrating the length of exon that circularizes into circRNAs; (**H**) Bar plot showing that one gene could generate multiple circRNAs.

Mice brain tissues with DMEM or RABV infection were subjected to RNA sequencing to further evaluate circRNAs expression patterns. In total, 30,985 distinct circRNAs candidates were identified in mice brain from two groups. Only 779 (2.51%) circRNAs were already annotated in circBase and 30,206 (97.49%) were not annotated, regarded as novel circRNA candidates (Figure 2A). The experimental and control groups shared 9021 circRNAs candidates, and 14,610 and 7354 circRNAs were specific to the RABV non-infection and infection groups, respectively (Figure 2B). The gene annotation revealed that these 30,985 distinct circRNAs originated from 6 genomic regions: 51.20%% for coding regions (exons, 3′UTR exon and 5′UTR exon), 16.77% for antisense, 11.55% for intronic, 7.21% for exon_intron, 7.14% for intergenic, 4.50% for 5′UTR, and 1.63% for 3′UTR (Figure 2C). Compared with the control group, RABV infection did not change the source type of circRNA, and circRNA were mainly derived from exons in both groups (Figure 2D,E). Consistent with previous studies in mice, human, and rat, circRNAs expressed in brain mainly derived from coding regions [39,40]. The majority of circRNAs (25,588/30,985, 82.58%) were less than 2000 nucleotides (nt) in length, and the others (5397/30,985, 17.42%) were longer than 2000 nt (Figure 2F), which is consistent with reports that the length of circRNAs tends to be shorter than 2000 nt [41,42]. CircRNAs derived from exons enclosed one or more exons, with the mean length of exon of one-exon circRNAs being longer than the mean of exon of multi-exon circRNAs (Figure 2G), which is in line with the observation that the length of circRNAs tends to be shorter than 2000 nt (Figure 2F) [43]. In addition, the circRNA derived from 6654 genes, and the amount of circRNA produced by each gene is different. Most genes (90.73%, 6037/6654) produced 1 to 10 circRNAs, while 617 genes generated more than 10 circRNAs (Figure 2H), which is far more than that of circRNAs derived from porcine intestinal epithelial cells [42]. A total of 174 out of 6654 genes (2.61%) generated ≥20 circRNA, and even of which, certain genes like RNA binding fox-1 homolog 1 (Rbfox1) and Kv channel interacting protein 4 (Kcnip4) could produce up to 100 circRNAs. The 174 of 6654 genes were analyzed by the Gene Ontology (GO) enrichment analysis and Kyoto Encyclopedia of Genes and Genomes (KEGG) pathway annotation. The top 20 GO terms and KEGG pathways are illustrated in Figure S1. GO analysis resulted that those 174 source genes were mainly engaged in processes related to cell morphogenesis, organization and development. The top 2 terms of the KEGG pathway annotation were cell adhesion of some molecules (CAMs) and MAPK signaling pathway, revealing that these 174 genes were mainly involved in inflammatory process. Notably, 5 pathways associated with synapse-related functions including CAMs, glutamatergic synapse, dopaminergic synapse, axon guidance, and GABAergic synapse showed enrichment in the top 20 list. Rbfox1 and Kcnip4 among the 174 genes were identified to regulate synaptic transmission [44] and be related to neurodevelopmental disorder [45]; indeed, for both of those genes, distinct isoforms encoded by multiple alternatively spliced transcript variants have been identified in humans [46,47]. These results suggest that genes involved in inflammatory process and associated with cell plasticity or synapse functions were active in generating circular transcripts in brain.

### 2.3. Identification of circRNAs Expression in RABV Infection

**Figure 3:**
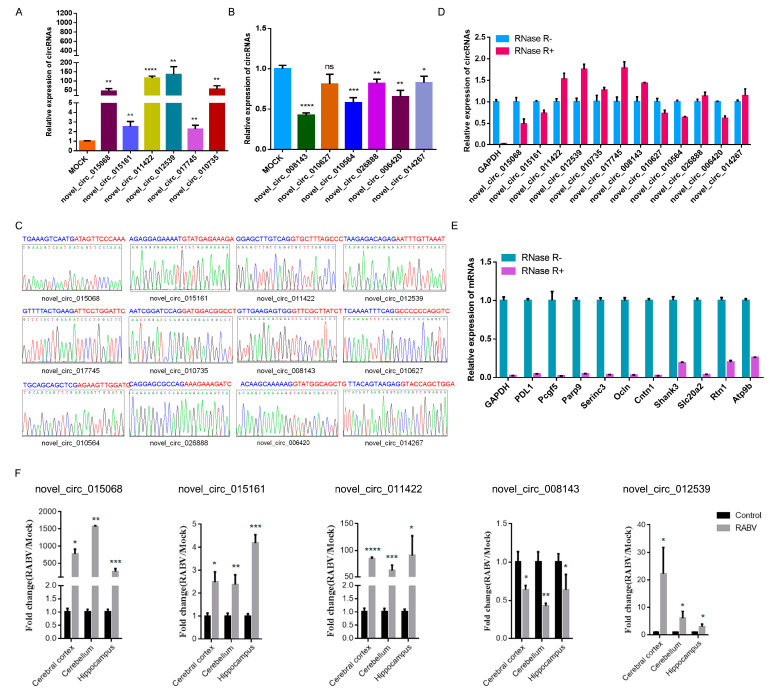
Validation of circRNAs. Expression changes of six upregulated (**A**) and six downregulated (**B**) circRNAs in RNA sequencing data were tested with divergent primers by RT-PCR; (**C**) Validation of back-splice junction sequences by Sanger sequencing; RNase-R resistance assay of 12 selected circRNAs (**D**) and corresponding linear transcripts (**E**); (**F**) Expression level of 5 circRNAs in the cerebral cortex, cerebellum, and hippocampus with or without RABV infection (*p* < 0.05, *; *p* < 0.01, **; *p* < 0.0005, ***; *p* < 0.0001, ****).

CircRNAs expression pattern was different in the control and experimental groups. A total of 636 differently expressed circRNAs were defined (*p* < 0.05 and fold change ≥2), of which 426 were significantly upregulated while 210 were significantly downregulated. We randomly selected 6 upregulated and 6 downregulated circRNAs to evaluate expression changes using RT-qPCR. We found that the expression trend of the 11 out of 12 circRNAs was consistent in RT- qPCR and RNA-seq analysis (Figure 3A,B); however, only the novel_circ_010627, which downregulated expression in RNA-seq analysis, showed no significant difference in RT-qPCR. Moreover, we confirmed the circRNAs sequence of head-to-tail junction site by Sanger sequencing (Figure 3C). The specific circular structure of circRNA led to its resistance to exonuclease Ribonuclease R (RNase R) digestion [48]. Our results showed that the abundance of linear transcripts significantly decreased after RNase R treatment, whereas the 12 selected circRNAs all showed apparent resistance to RNase R digestion (Figure 3D,E), further confirming that the randomly selected circRNAs in RNA-seq displayed circularity. For 5 circRNAs, we also examined the expression changes of circRNAs in cerebral cortex, cerebellum, and hippocampus with or without RABV infection by RT-qPCR. The expression pattern of novel_circ_015068, novel_circ_015161, novel_circ_011422, novel_circ_012539, and novel_circ_008143 in the specific brain regions was consistent with the expression pattern in total brain (Figure 3F). These results indicate that RABV infection shifts the circRNAs expression in mice brain and that the nervous system showed extensively differential expression of circRNAs in RABV infection.

### 2.4. Expression Correlation of circRNAs and mRNAs

**Figure 4:**
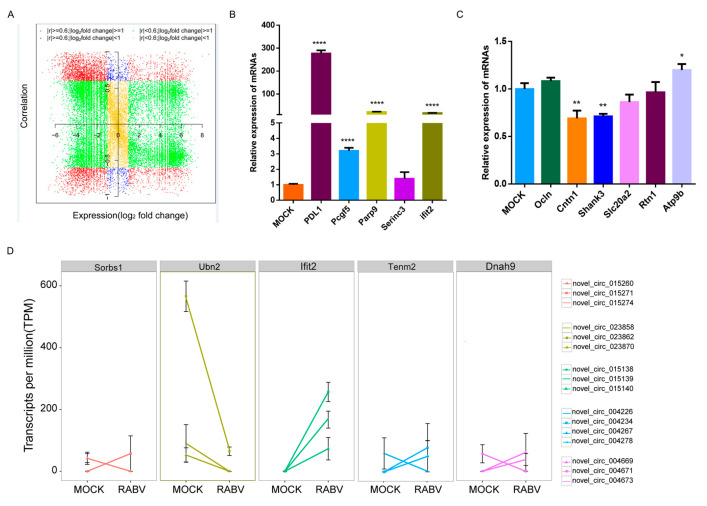
Expression correlation between circRNAs and linear counterparts. (**A**) The expression correlation between circRNAs and corresponding linear transcripts. The circRNAs are colored according to the Pearson correlation coefficient (r) and their expression pattern in RABV infection; (**B**) Expression changes of linear transcripts of six upregulated circRNAs and six downregulated circRNAs (**C**) were tested by RT-qPCR; (**D**) Variable expression patterns of circRNAs derived from Ubn2, Ifit2, Sorbs1, Tenm2, and Dnah9 (*p* < 0.05, *; *p* < 0.01, **; *p* < 0.0001, ****).

To gain a better understanding of the expression pattern and function of circRNAs in RABV infection, the rRNA-depleted RNA samples were sequenced. A total of 21,923 mRNA were identified, 4437 mRNAs of which were differentially expressed between the two groups, and the number of differentially expressed mRNAs far exceeds that of circRNAs. Interestingly, the number of upregulated mRNAs was excessive compared to that of the downregulated mRNAs (3621 vs. 816), which is consistent with that of circRNAs. Next, we explored the correlation between the expression of circRNAs and source gene mRNA transcripts by calculating the Pierce correlation coefficient. We found that the expression of 1467 circRNAs was positively correlated with their corresponding linear transcripts while 928 circRNAs were negatively correlated (Figure 4A). Furthermore, the expression pattern of linear transcripts was examined by qPCR. We found that genes such as PD-L1, Pcgf5, and Parp9 showed similar increased fold change in their circRNAs (novel_circ_015068, novel_circ_015161, and novel_circ_011422) and mRNAs expression in RABV infection. Whereas, the Atp9b gene had decreased novel_circ_014267 expression while increased mRNA expression in RABV infection. In contrast, Serinc3 significantly increased the novel_circ_017745 expression, and genes such as, Ocln, Slc20a2, and Rtn1 had significantly decreased circRNAs (novel_circ_008143, novel_circ_026888, and novel_circ_006420) expression, while their mRNA expression remained unchanged in RABV infection. Cntn1 showed unchanged in novel_circ_010627 expression in RABV infection, while the mRNA was significantly decreased in RABV infection (Figure 4B,C). Together, circRNAs expression variation can be related or be independent of their host genes.

As shown in Figure 2H, one gene could generate more than one circRNA. Interestingly, circRNAs originating from the same gene could show a diversified expression pattern (Figure 4D). For example, circRNAs generated from Ubn and Ifit2 showed the same expression trend, all showing upregulated or downregulated expression. Whilst circRNAs arose from Sorbs1, Tenm2 and Dnah9 showed converse expression trend, certain circRNAs showing upregulation while others showed downregulation. The expression diversifications of the individual circRNA and linear host transcripts may be relevant to different biogenesis mechanisms and biological functions of linear and circular transcripts.

### 2.5. Functional Enrichment Analysis of Host Genes of Differentially Expressed circRNAs

**Figure 5:**
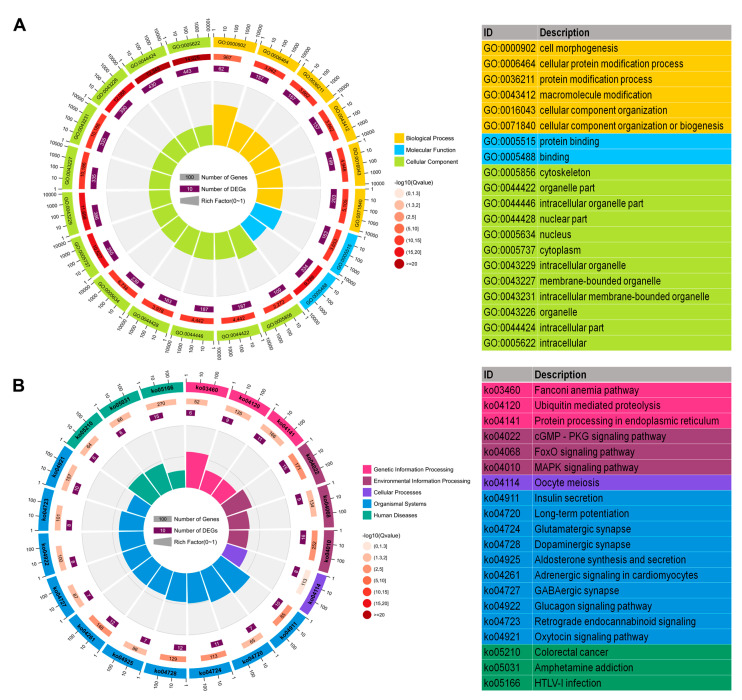
GO and KEGG enrichment analysis. The circular graph shows the top 20 enriched GO terms (**A**) and KEGG pathways (**B**) of source genes of 636 differentially expressed circRNAs.

**Figure 6:**
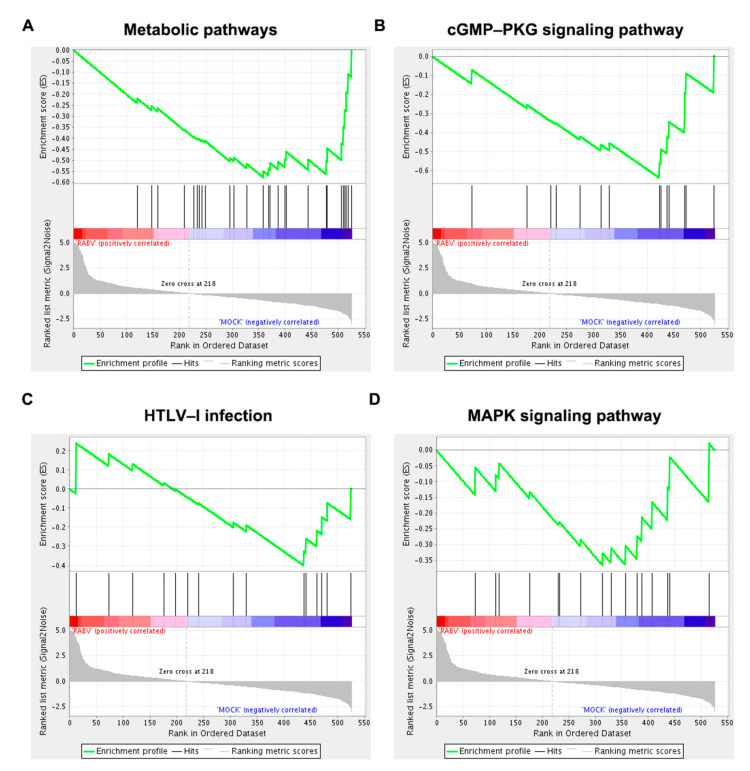
Representative 4 significantly enriched gene sets from GSEA analysis. Source genes of significantly differentially expressed circRNAs were specifically engaged in (**A**) metabolic pathways, (**B**) the cGMP–PKG signaling pathway, (**C**) HTLV-I infection, and (**D**) the MAPK signaling pathway.

To gain insight into the potential function of host genes of differentially expressed circRNAs, we conducted GO, KEGG, and Gene Set Enrichment Analysis (GSEA). GO analysis indicated that source genes of circRNAs were mainly engaged in cellular components such as intracellular part, intracellular, intracellular organelle, cytoplasm, and organelle (Figure 5A). The top 5 KEGG signaling pathways included the cGMP–PKG signaling pathway, insulin secretion, dopaminergic synapse, glutamatergic synapse, and MAPK signaling pathway (Figure 5B). Interestingly, functions related to glucose metabolism and synaptic functions were significantly enriched (Figure 5B). Functional terms related to synaptic functions were dopaminergic synapse, glutamatergic synapse, long-term potentiation, retrograde endocannabinoid signaling and GABAergic synapse. Insulin secretion, the glucagon signaling pathway, and aldosterone synthesis and secretion relate to glucose metabolism. Moreover, GSEA showed that host genes were closely associated to metabolic pathways, the cGMP–PKG signaling pathway, HTLV-I infection, and MAPK signaling pathway. Except for the metabolic pathways, the other three pathways were also found in the KEGG analysis (Figure 6).

### 2.6. Interaction Network of circRNA–miRNA–mRNA

**Figure 7:**
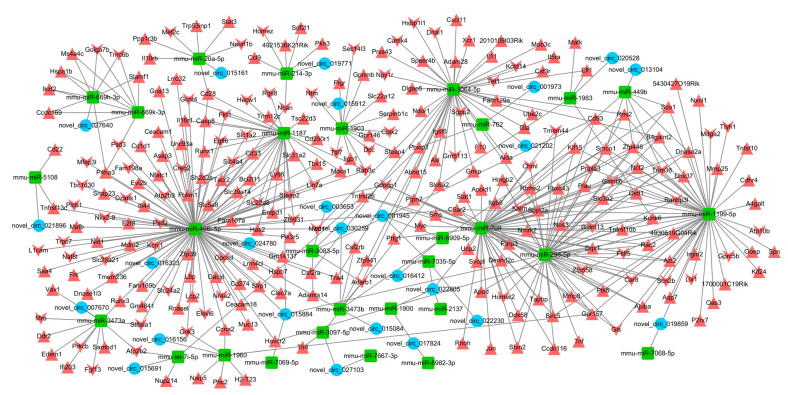
The circRNA–miRNA–mRNA interaction network. The blue circles represent circRNAs; the green rectangles represent miRNAs; the red upward triangles represent upregulated mRNAs, while the red downward triangles represent downregulated mRNAs. This network was visualized using Cytoscape software.

These circRNA–miRNAs were predicted to interact with strong correlation (*p* < 0.05 and score >1.5) and genes altered the expression of mRNAs in RABV infection (*p* < 0.05 and fold change >2) identified in this study were included to build the circRNA–miRNA–mRNA network (Figure 7). The ceRNA network comprised 25 circRNAs (17 upregulated and 8 downregulated circRNAs), 29 miRNAs and 264 mRNAs (186 upregulated and 78 downregulated mRNAs). Novel_circ_015884 and novel_circ_022805 possessed the most miRNA binding sites with four circRNA–miRNA interactions. Among all the circRNA–miRNA pairs and miRNA–mRNA pairs, mmu-miR-466i-5p was the most abundant, which was predicted to interact with 3 upregulated circRNAs (novel_circ_015884, novel_circ_016323 and novel_circ_024780) and 95 mRNAs. A total of 36 circRNA–miRNA pairs, and 416 miRNA–mRNA pairs were identified in the network, which means host circRNAs may regulate RABV infection through modulating genes expression via interacting with miRNAs.

## 3. Discussion

CircRNAs are widely expressed in different tissues, whereas the brain especially expresses abundantly circRNAs in drosophila, mouse, and human due to the high speed of transcription and alternative splicing rates of neurons [39,49,50]. CircRNAs are found to regulate microglial activation [51], synaptic gene expression [52], and neurological disorders [5,23,35,39]. Rabies is a high lethal encephalomyelitic disease induced by RABV. However, whether circRNAs participated in RABV infection and the regulatory mechanism of circRNAs is still scant. In this study, RABV-infected and DMEM-infected mice brains were subjected by RNA sequencing and 30,985 circRNAs candidates were identified in two groups. Among these, 14,610 and 7354 circRNAs were expressed specifically to the control and experimental groups, indicating that the expression of certain circRNAs was inhibited and other circRNAs were induced on RABV infection. Together with the observation that 97.5% of all the identified circRNAs candidates were newly identified, the circRNAs population is thus still largely unknown and certain circRNAs may be RABV specific. The basic characteristics of circRNAs, including the derived genomic region, the length of circRNAs and exons comprising circRNAs, were consistent with previous studies [39,40,41,42,43]. The number of produced circRNAs from individual gene is variable and genes that generated ≥20 circRNAs were most enriched in two inflammatory process, CAMs and MAPK signaling pathway, in the KEGG analysis. Genes active in producing circRNAs were also related to cell plasticity and synapse function as shown in both GO and KEGG enrichment analysis (Figure S1), which can partially explain in previous studies where circRNAs were abundantly enriched in synapses of brain [39]. Therefore, it is easy to suspect that host genes that mediate inflammatory process and regulate cell plasticity or synapse function tend to produce multiple circRNAs.

Recently, transcriptome profiling revealed the differential expression pattern of long noncoding RNAs (lncRNAs) and miRNAs in RABV infection, and their involvement in multiple immunity-related signal pathways [53,54,55,56,57]. Tu et al. reported that miR-455-5p promotes viral replication via the SOCS3/STAT3/IL-6 pathway [58]. A recent study reported that lncRNA EDAL degrades EZH2 by binding to it, thereby reducing H3K27me3 levels to retain replication of multiple neurotropic viruses [59]. Thus, host-derived non-coding RNAs are critical in regulating antiviral response against viral infections. In this study, RABV infection significantly altered the expression pattern of 636 circRNAs: 426 were significantly upregulated and 210 downregulated circRNAs (fold change ≥2 and *p* value < 0.05). The relative expression levels of 12 circRNAs in total brain tissues were further tested by RT-qPCR. We found that 90% of randomly selected circRNAs expression was consistent with that of RNA sequencing (Figure 3A,B). RNAse R-resistant assay and Sanger sequencing were further validated in the circular structure of these randomly selected circRNAs (Figure 3C–E). Five circRNAs were further validated in their expression in the cerebral cortex, cerebellum, and hippocampus (Figure 3F), and the results confirmed that circRNAs expression profiling was perturbed in RABV infection. Given that the exon sequences of circRNAs junctions were highly conserved [40], we thus hypothesize that these differentially expressed circRNAs potentially function in RABV infection, and the mechanism in modulating RABV infection needs further exploration.

RABV is a highly neurotropic virus with virus propagation relying on synapses [60]. Five out of the top 20 KEGG pathways were related to synaptic functions, including dopaminergic synapse, glutamatergic synapse, long-term potentiation, retrograde endocannabinoid signaling and GABAergic synapse (Figure 5B). Genes relevant to glutamatergic synapse, dopaminergic synapse and GABAergic synapse induced differentially expression of circRNAs in RABV infection, and were also revealed to produce circRNAs actively (Figure S1), indicating that source genes of differentially expressed circRNAs are mainly related to synaptic function and these genes may regulate synaptic function by producing circRNAs and thus regulate viral transport in CNS. Metabolism was another significantly enriched function in both KEGG and GSEA analysis, such as insulin secretion, glucagon signaling pathway, and aldosterone synthesis and secret, metabolic pathways, and the cGMP–PKG signaling pathway. Emerging evidence indicates that insulin signaling is engaged in various viral infections, such as those caused by Chronic hepatitis C virus (HCV) [61], Zika virus [62], and influenza virus [63]. Hyperinsulinemia induced by viral-induced insulin resistance directly stimulates the function of CD8^+^ effector T cells to enhance antiviral immunity [64], which provides evidence that metabolic abnormalities induced by endocrine dysfunction is a physiological mechanism of the immune system against virus infections. Insulin secretion pathway was a main enriched pathway revealed by the circRNA profiling in this study, thus insulin secretion could be a vital strategy facilitating RABV infection.

Multiple studies have validated circRNAs-regulated target transcripts function by serving as miRNA sponges on virus infection. For example, circ-Vav3 increases the expression of YAP1 by suppressing the activity of gga-miR-375 to promote liver tumors induced by avian leukosis virus subgroup J [37]. We thus build a comprehensive co-expression network of circRNA-miRNA-mRNA (Figure 7). Among all the circRNA–miRNA pairs and miRNA–mRNA pairs, mmu-miR-466i-5p was the most abundant, which was predicted to interact with 3 upregulated circRNAs (novel_circ_015884, novel_circ_016323 and novel_circ_024780) and 95 mRNAs. Moreover, many of these mRNAs binding mmu-miR-466i-5p were upregulated, induced by RABV infection (red triangles in Figure 7), several of which were proven to be involved in microbes infection and immune response. For example, CEACAM1 is a receptor of murine hepatitis virus [65] and several bacteria; also the oligomerization of CEACAM1 mediate immune envision by inhibiting NK and T cell activity [66]. Mafb suppresses production of type I IFN by CD14+ monocytes, impairing antiviral responses [67]. Therefore, the miR-466i-5p activity was restricted by novel_circ_015884, novel_circ_016323 or novel_circ_024780, resulting in the increased level of target mRNAs such as CEACAM1 or Mafb and then perhaps contribute to the immune escape response of RABV. A previous report demonstrated that B7-H1 (also known as PD-L1 or CD274) protein production was highly dependent of TLR3 signaling in RABV infection and the upregulated B7-H1 inhibited the CD8^+^ T cell proliferation and finally performed a contribution effect on viral immune evasion [11]. We also validated that the increased expression of B7-H1 mRNA and B7-H1 was predicted to be one of the target genes of mmu-miR-466i-5p, suspecting that novel_circ_015884, novel_circ_016323 or novel_circ_024780 restrict mmu-miR-466i-5p activity through regulating B7-H1 expression and then modulate RABV replication. Interestingly, novel_circ_015068, the only circRNA candidate originated from B7-H1 in this study, showed significantly upregulation in RABV infection. Thus, it is likely that, in addition to protein production, gene B7-H1 can also regulate RABV infection by generating circular transcripts However, whether novel_circ_015068 is synergistic, antagonistic, or irrelevant in RABV infection with B7-H1 protein is unknown; however, the relevance of circular transcripts and protein in regulating viral replication is worth investigation. Another miRNA, mmu-miR-762, was predicted to interact with novel_circ_001973 in the ceRNA network. miR-762 expression level was increased in Hepatitis C patients serum with direct-acting antiviral therapies after the clearance of the virus, and overexpression of miR-762 decreased the replication of viral RNA in vitro [68], indicating that the novel_circ_001973 may facilitate RABV infection via sponging with miR-762. Still, many circRNAs, miRNAs and mRNAs in the network have not been studied and the function mechanism that they regulate virus replication requires further exploration. Many circRNAs are not in the ceRNA network graph, indicating that these circRNAs may modulate virus replication through other mechanisms, such as sequestering proteins, regulating gene transcription or translating into proteins.

In summary, we characterized the transcriptomic landscape of circRNAs and identified differential expression patterns of circRNAs in RABV infection. Enrichment analysis revealed differentially regulated circRNAs mainly originated from genes associated with cell plasticity and synapse function. Both KEGG and GSEA analysis showed that these host genes were involved in the cGMP-PKG and MAPK signaling pathway, and HTLV-I infection. Finally, ceRNA network provided a new vision of the pathogenesis of RABV from the perspective of circRNAs.

## 4. Materials and Methods

### 4.1. RABV Infection

The Challenge Virus Standard (CVS) -11 strain of RABV was kept in our laboratory and the virus titer was determined by the Reed–Muench method [69]. All experiments involving live viruses were carried out under Biosafety Level 2+ containment following the Institutional Animal Care and Use Committee of Nanjing Agricultural University, Nanjing, China (No. SYXK2017-0007, February 2017, Institutional Animal Care and Use Committee of Nanjing Agricultural University) and met the standard of the International Guiding Principles for Biomedical Research Involving Animals. Adult C57BL/6 mice were obtained from Nanjing Mutu Technology Co., Ltd., Nanjing, China. Mice were separated into the experimental group and the control group randomly, each with 9 mice. Both groups were intranasally inoculated with CVS-11 strain or only equal volume of DMEM. We monitored these mice every day for clinical symptoms observation. Once the infected mice showed obvious weight loss and clinical signs, both groups of mice were intraperitoneally euthanized with 2.5% Avertin (150 µL/10 g). The blood was washed with physiological saline by the transcardial perfusion method. Then brains were carefully collected for further validation of RABV infection and RNA sequencing.

### 4.2. RNA Extraction and Quantitative Real-Time PCR

Brain tissues were treated using TRIzol (Invitrogen, Carlsbad, CA, USA), subsequently for total RNA extraction. Equal concentrations of total RNA (1 μg) were subjected to removal of genomic DNA with DNAse I (Thermo Scientific, Waltham, MA, USA), and then first-strand cDNA was synthesized using the RevertAid™ First Strand cDNA Synthesis Kit (Thermo Scientific) with random hexamers. Q PCR was conducted to detect the RABV nucleoprotein gene (N), circRNAs and mRNAs using AceQ^®^ qPCR SYBR^®^ Green Master Mix (Vazyme, Nanjing, China). In brief, samples were incubated at 95 °C for 10 min, followed by 40 cycles at 95 °C for 15 s and finally at 60 °C for 30 s. RABV-N-specific primer set and GAPDH-specific primer set were: 5′-TTGGCCGGAACCTACGACAT-3′ and 5′-AGTATTGCTTCCCTTGCGGTG-3′ for RABV-N; 5′-AGGTCGGTGTGAACGGATTTG-3′ and 5′-TGTAGACCATGTAGTTGAGGTC-3′ for GAPDH. Each target was detected in triplicate and normalized to that of GAPDH using the 2^−ΔΔCt^ method. Table 1 and Table 2 list the primer sequences for detecting circRNA and corresponding mRNA.

### 4.3. Immunohistochemical (IHC) Analysis

The paraformaldehyde-fixed brain tissues were embedded with paraffin. After deparaffinization and rehydration in xylene and ethanol, sections were incubated with monoclonal antibody 1C5 RABV-N protein (Hytest) as the primary antibody overnight at 4 °C. After 3 washes, the sections were incubated with HRP-conjugated anti-mouse IgG as the secondary antibody (MAIXIN. Bio, Fuzhou, China). Lastly, 3,3′-diaminobenzidine was used to visualize virus particles.

### 4.4. RNA Sequencing Analysis

For circRNAs sequencing, total RNA was digested with RNase R to degest linear RNAs and was further purified by using the RNeasy MinElute Cleanup Kit (Qiagen, Hilden, Germany). VAHTS Total RNA-seq (H/M/R) Library Prep Kit for Illumina was used for constructing the strand-specific library following the manufacturer’s instructions. For mRNA sequencing, mRNA was enriched using Oligo (dT) beads and the next process was similar with circRNAs sequencing. After sequencing, the clean reads were mapped to reference genomes by TopHat2 [70] (version 2.0.3.12). Those unmapped reads to the genomes were abandoned, and these could not be mapped and were then collected to analyze unique anchor positions within the splicing site. Candidate circRNAs were subjected to blast in the circBase database [71] for annotation, and unannotated circRNAs were recognized as novel ones. For mRNAs, the unmapped reads to reference genomes by TopHat2 [70] were realigned with Bowtie2 [72]. The unmapped reads were then split into smaller segments for further gene annotation [73]. The DESeq2 package [74] and the edgeR package (http://www.r-project.org/, accessed 21 May 2019) were applied to analyze differentially expressed circRNAs and mRNAs, respectively. Candidates were considered significantly differentially expressed ones if a fold change ≥2 and a *p* value < 0.05.

### 4.5. Functional Enrichment Analysis

All genes that generated ≥20 circRNA candidates or source genes of significantly differentially expressed circRNAs were subjected to the Gene Ontology (GO) database or Kyoto Encyclopedia of Genes and Genomes (KEGG) database. All the functional enrichment analysis were carried out using hypergeometric tests. The calculated *p* value was corrected by the false discovery rate (FDR), then GO terms or KEGG pathways were recognized as significantly enriched ones when FDR ≤ 0.05.

### 4.6. Gene Set Enrichment Analysis (GSEA)

Gene Set Enrichment Analysis (GSEA) was subjected to software GSEA and MSigDB [75] to analyze whether source genes of significantly differentially expressed circRNAs were specifically enriched in certain pathways. In Brief, gene expression matrix and rank genes were performed by SinaltoNoise normalization method. Enrichment scores and *p* value was calculated in default parameters.

### 4.7. Integrated Analysis of circRNAs-miRNAs-mRNAs

The Mireap, Miranda (v3.3a) and TargetScan (Version:7.0) software packages were used to predict potential miRNAs targets of 636 differentially expressed circRNAs, and subsequently miRTarBase (v6.1) was used to get the validated miRNA—mRNA interactions. CircRNA-miRNA pairs with strong correlation (*p* value < 0.05 and score >1.5) and mRNAs altered by RABV infection (*p* value < 0.05 and fold change >2) were finally included to build the circRNA-miRNA-mRNA network, visualizing by Cytoscape v3.7.1.

## Figures and Tables

**Table 1:** Primers used for validating the expression of circRNAs.

Oligonucleotide Sequences	5′–3′
novel_circ_015068	TGCATAATCAGCTACGGTGGTG
	TGGAGCCGTGATAGTAAACGC
novel_circ_017745	TTGGTTCTTCAAAATCGCTGCC
	TCTGCCTTTGTATCAACCATCTTG
novel_circ_015161	GCTGTGCAATGGTGAAATTATGG
	CACTGAGCATCACTCACTCCTTT
novel_circ_011422	GCTGTGTGACGTTCAGGAAGAA
	TTCAAGGCCGCCTCTTCTTTG
novel_circ_012539	TTGGAATGTTGGACTCCTTGAGA
	CCTTCATCCAACCAGGCACTT
novel_circ_010735	CCACAGCCTTGCTTGTGAAA
	CTGTTTCCTCTACTGATGGTGGG
novel_circ_008143	ACCCGAAGAAAGATGGATCGG
	CAGGCTCCCAAGATAAGCGA
novel_circ_010627	ACGGAGAGTATGTTGTCGAGGT
	AACCACGGCTCCAAGTCAGT
novel_circ_010564	TGGACCCTGCTAAGAAGTCACC
	TCTGCAATGTCCGGCCTCA
novel_circ_026888	TGCCTCTGTTTTATGCTGCTACC
	TGGTTACGGTCAGTCAGTGGT
novel_circ_006420	AGTCAAAGTCCCACGGCCAC
	TAGTGTTGAAGAGGGAGAGTGGT
novel_circ_014267	TTTCGACGTTTTCAGCGAGACA
	GCCTTCTTCAGACATCATCAGTGG

**Table 2:** Primers used for validating the expression of linear transcripts of circRNAs.

circRNAs	Source Gene	Oligonucleotide Sequences (5′–3′)
novel_circ_015068	PD-L1	CCTGCTGTCACTTGCTACGG
		TCCAGCTCCCGTTCTACAGG
novel_circ_015161	Pcgf5	TGCCCTTCTGCTACTGACCA
		GGCAAGCGGAACACTGAGAA
novel_circ_011422	Parp9	ACAGGGAAGAGCAAAGGCGA
		GTGGCCTGTTTCGGGTGATG
novel_circ_012539	-	

novel_circ_017745	Serinc3	AGGAACATCAGCCTCGCTCT
		GGACCGCTCAGGTTCATTGG
novel_circ_010735	-	

novel_circ_008143	Ocln	GGGAAAGCAGGAAAGGGCAA
		CTGACCCAGTCCTCCTCCAG
novel_circ_010627	Cntn1	GGGCTGGGCATGACAAAGAA
		TGGGTGTCGGGAAGAAGGTT
novel_circ_010564	Shank3	TTCCTCTCTGTGGGTGCCAT
		CAGGGGAGGGGAGTAGCAAA
novel_circ_026888	Slc20a2	GGGTTTGGGGCAGAAGAGTG
		TGTTGGAGGCAATCACCACG
novel_circ_006420	Rtn1	GGGTTTGGCACATCCCCTAA
		TTCGCTACTCCCAAGCCTGT
novel_circ_014267	Atp9b	ACAGTTCACGGGCTGGTTTC
		ATGGGGGCATCTCGAAGTCA

## Data Availability

Not applicable.

## Supplementary Materials

The following are available online at https://www.mdpi.com/article/10.3390/ijms22126537/s1, Figure S1. GO and KEGG enrichment analysis of host genes that generated more than 20 circRNAs.
