# Supplementary figures and images for: Expression Profiling and Bioinformatics Analysis of CircRNA in Mice Brain Infected with Rabies Virus
Source: Int J Mol Sci. 2021 Jun 18;22(12):6537. doi: 10.3390/ijms22126537 (PMC8234020; doi:10.3390/ijms22126537)

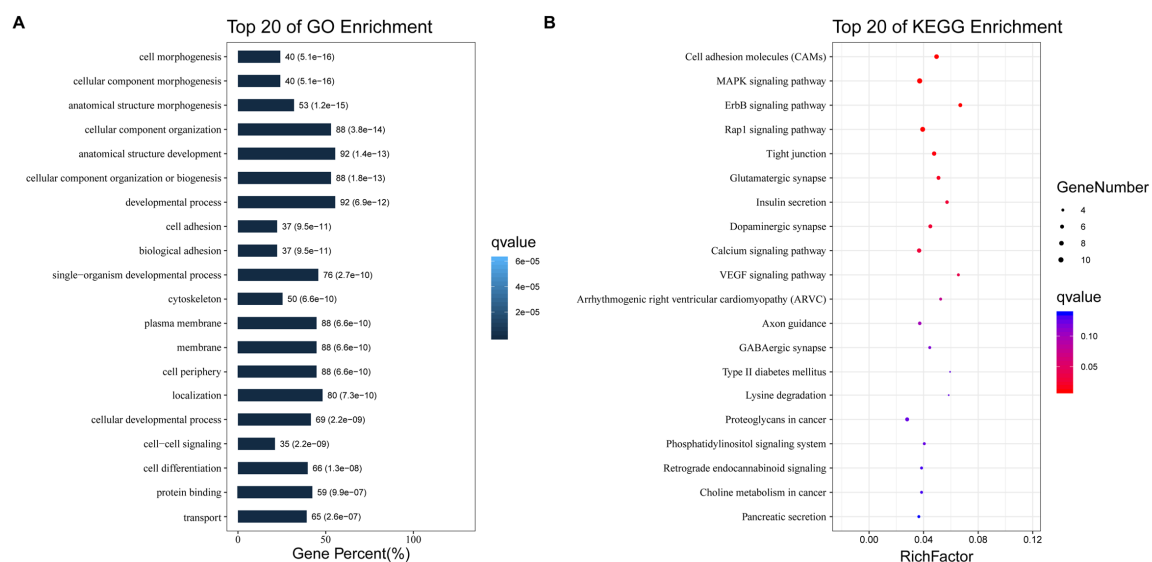

**Figure S1.** GO (A) and KEGG (B) enrichment analysis of host genes that generated more than 20 circRNAs.

Supplement: Supplementary file 1 [file ijms-22-06537-s001.zip › ijms-1230814-supplement.pdf]
